# Screening for depression in primary care: a Rasch analysis of the PHQ-9†

**DOI:** 10.1192/pb.bp.114.050294

**Published:** 2016-10

**Authors:** Mike Horton, Amanda E. Perry

**Affiliations:** 1 University of Leeds, UK; 2 University of York, UK

## Abstract

**Aims and method** To explore the modern psychometric properties of the Patient Health Questionnaire (PHQ-9), we used the Rasch analysis in a sample of 767 primary care patients with depression.

**Results** The analysis highlighted dependency issues between items 1 and 2 (‘Little interest or pleasure in doing things' and ‘Feeling down, depressed, or hopeless’), and items 3 and 4 (‘Trouble falling or staying asleep, or sleeping too much’ and ‘Feeling tired or having little energy’). Items 1 and 2 displayed an over-discrimination, suggesting their potential redundancy within the complete item set.

**Clinical implications** In its current format the PHQ-9 displays some problems with regard to its measurement structure among a sample of primary care patients. These problems can be addressed by removing potentially redundant items to deliver a stable screening tool. The results also lend support for the PHQ-2 to be used as a screening tool in a primary care setting.

Depression is a common mental health problem, with 10% of the adult population affected at any one time.^1–4^ The Patient Health Questionnaire-9 (PHQ-9)^5^ was designed as a case-finding instrument for depression in primary care and has been tested in a range of populations.^6–9^ The shorter version, Patient Health Questionnaire-2 (PHQ-2)^10^ contains two key items of the PHQ-9 and is now well established as a sufficient measure of screening for depression.^11,12^

The PHQ-9 was developed and assessed using traditional psychometric attributes that are underpinned by classical test theory (CTT), which is the mostly widely used method for constructing and evaluating rating scales. Despite its prevalence, CTT has some limitations, including that ordinal data are treated as interval level, the evaluations of scales are sample dependent, and the assumptions of CTT cannot be formally tested.^13^

Recent advances in the application of modern psychometric methodology (e.g. Rasch modelling)^14^ provide a useful supplement to traditional psychometric methods. Rasch analysis is the formal testing of an outcome scale against a mathematical measurement model that operationalises formal measurement.^15^ The Rasch model shows what should be expected in responses to items if interval measurement is to be achieved, and provides a proper method for non-linear transformation of ordinal raw scores to interval measures.^15,16^

The PHQ-9 has not been evaluated using Rasch analysis on a sample of adults with depression in primary care services. Furthermore, the scale has not been fully examined in terms of the underlying assumptions of local independency. We have set out to address this.

## Method

### Participants

A cross-sectional sample of 767 participants were recruited as part of the Randomised Evaluation of the Effectiveness and Acceptability of Computerised Therapy (REEACT) trial (www.york.ac.uk/healthsciences/research/mental-health/projects/reeact/). Participants were included if they were: (a) adults aged 18 years and above; (b) diagnosed with depression; and (c) not currently in receipt of computerised cognitive-behavioural therapy (CBT) or specialist psychological therapy. Patients were excluded if they were: (a) actively suicidal; (b) experiencing psychotic symptoms; (c) were diagnosed previously with post-natal depression; (d) recently bereaved; and (e) had psychotic depression. PHQ-9 data are available for all 767 screened participants, but demographic information is only available for the participants who were entered into the study (*n* = 695). Of those, a third were male (*n* = 229) and 95% (*n* = 657) were of White ethnic origin. The mean age was 39.8 years (s.d. = 12.7; range 18.5–76.2).

### Measure

The PHQ-9 is a nine-item measure of depressive symptoms containing four somatic items (items 3, 4, 5 and 8) and five items relating to thoughts and feelings linked to depressed mood (items 1, 2, 6, 7 and 9). Respondents are asked to report on the frequency of their symptoms during the past 2 weeks using four response categories: 0 (not at all), 1 (several days), 2 (more than half the days) and 3 (nearly every day). The total score ranges from 0 to 27, and the recommended cut-off point to classify clinical depression is a score of 10. The PHQ-9 has consistently demonstrated robust psychometric properties, reliability and validity in adult populations.^17–19^

### Procedures

Administration of the PHQ-9 was conducted at baseline (prior to randomisation), and at 4, 12 and 24 months post-randomisation as part of a battery of tests for the larger REEACT study during the recruitment period. Trained interviewers at each of the four trial sites (York, Manchester, Bristol and Sheffield) read or asked participants to self-report their responses on the PHQ-9 items and recorded the responses. The data included all screened participants (*n* = 767).

### Rasch analysis

Analysis was conducted on the entire baseline sample (*n* = 767) using RUMM 2030 software (www.rummlab.com.au) using the partial credit (unrestricted) parameterisation of the model.^20^ Assessing the internal construct validity of the PHQ-9 involved investigating the individual item thresholds, the overall scale fit, individual item fit to the measurement model and issues relating to the local independency assumptions. The response structure across all items was investigated to assess whether it was working as intended. This was explored by assessing the thresholds at the cross-over points between adjacent response categories, which should remain ordered in a logical pattern.^16^ The overall scale fit statistics provide a summary measure of how the scale conforms to Rasch model expectations. Reliability indices are delivered in the form of a person separation index (PSI) and Cronbach's alpha. Analysis was also conducted at the individual item level, where adequate fit is indicated by non-significant chi-squared test values and *z*-standardised fit residual statistics within +/−2.5.^21^ High positive fit residual values indicate a misfit to model expectations, while high negative fit residuals may suggest item redundancy.^22^

A residual correlation matrix was used to explore the extent of dependency between items within the scale. Dependency occurs when the response to one item has a direct effect on the response to another item within the scale, conditional on the level of depression. If present, this is liable to artificially inflate reliability indices and to create spurious multidimensionality within an item set.^22,23^ Residual correlations above a value of 0.2 indicate a significant level of dependency between items,^24^ although values above 0.1 have been used to identify potential response dependency.^23^ Response dependency can be accounted for by grouping the dependent items together into ‘sub-tests’ within the analysis framework.

The unidimensionality of the scale was assessed using a *t*-test procedure^25^ whereby the percentage of significant individual *t*-tests should not be above 5%. In practice, the lower bound of a binomial confidence interval should overlap the 5% level to indicate an acceptable absence of multidimensionality within the scale.^15^

The targeting of a scale to the study sample is assessed by investigating the relative logit locations of the item threshold distribution and person location distribution. The primary purpose of scales may differ, but for a well-targeted measure, the mean person location should not deviate too much from the mean item difficulty (fixed at 0 logits).^15^ This distribution is also reflected in the Person-Item Threshold Distribution plot available in the RUMM2030 software.

The second element of the analysis involved the exploration of ways to account for any misfit found within the scale and offers further insight into the contributions of each individual item. Iterations of the analyses involved a combination of item removal or sub-testing to account for response dependency.

## Results

### Part one: PHQ-9 assessment

The sample of 767 contained a full range of scores (0–27) with no missing data (median 16; interquartile range 7). Over 90% (*n* = 695) scored above the cut-off point for clinical depression (i.e. a score of 10 or over).

#### Thresholds of individual items

None of the items displayed reversed/disordered thresholds, meaning that the original PHQ-9 response structure appears to be functioning as intended. The threshold marking the lower boundary of the scale is the transition from response category 0 (not at all) to response category 1 (several days) on item 2. The threshold marking the upper boundary of the scale is the transition from response category 2 (more than half the days) to category 3 (nearly every day) on item 9.

#### Initial fit to the Rasch measurement model

All iterations of the analysis are summarised in Tables 1a and 1b.

**Table 1a:** Summarised analysis results of the PHQ-9

Analysis	Analysis summary	Item location Mean (s.d.)	Person location Mean (s.d.)	Item fit residual Mean (s.d.)	Person fit residual Mean (s.d.)	Chi square interaction Value (d.f.) *P*	PSI with extrms/ no extrms	Alpha
Initial	Nothing – initial	0 (0.821)	0.465 (1.038)	0.107 (2.01)	−0.23 (1.04)	189.5 (81) 0	0.793/0.766	0.795

A	Item 2 removed from initial as part of dependent pair	0 (0.803)	0.368 (0.97)	0.162 (1.12)	−0.23 (0.98)	109.9 (56) <0.0001	0.751/0.719	0.756

B	Item 1 removed from initial as part of dependent pair	0 (0.849)	0.41 (0.997)	0.123 (1.57)	−0.23 (0.98)	155.6 (72) 0	0.757/0.723	0.763

C	Items 1 & 2 sub-tested to account for dependency	0 (0.803)	0.37 (0.959)	−0.03 (1.84)	−0.25 (0.98)	115.1 (72) 0.001	0.775/0.745	0.77

D	Items 1 & 2 and 3 & 4 sub-tested to account for dependency	0 (0.797)	0.26 (0.933)	0.015 (1.57)	−0.24 (0.95)	97.04 (63) 0.004	0.768/0.735	0.756

E	Items 1, 2 & 6 and 3 & 4 sub-tested to account for dependency	0 (0.802)	0.165 (0.87)	−0.07 (1.81)	−0.24 (0.88)	72.81 (54) 0.045	0.75/0.713	0.709

F	Item 2 removed and items 3 & 4 sub-tested to account for dependency	0 (0.798)	0.259 (0.944)	0.252 (0.52)	−0.22 (0.95)	87.25 (56) 0.005	0.745/0.707	0.733

G	Items 1 & 2 removed	0 (0.83)	0.301 (0.927)	0.172 (0.96)	−0.24 (0.93)	102.2 (63) 0.0013	0.704/0.669	0.717

H	Items 1 & 2 removed and Items 3 & 4 sub-tested for dependency	0 (0.809)	0.163 (0.892)	0.299 (0.47)	−0.23 (0.9)	81.82 (54) 0.0086	0.692/0.649	0.686

PHQ2	PHQ-2 items only	0 (0.42)	1.3 (2.11)	0.47 (0.31)	−0.49 (0.85)	16.93 (6) 0.0095	0.606/0.45	0.757

**Table 1b:** Summarised analysis results of the PHQ-9

		Unidimensionality *t*-tests (CI)			
Analysis	Analysis summary	Significant tests *n* (%)^a^	Lower- bound 95% CI	Misfit items, *n* (χ^2^ or fit residual)	Response- dependent items^b^
Initial	Nothing – initial	56 (7.40)	5.80%	Items 1, 2, 3	Items 1 & 2, 2 & 6, 3 & 4

A	Item 2 removed from initial as part of dependent pair	37 (4.90)	–	Item 4	Items 3 & 4

B	Item 1 removed from initial as part of dependent pair	42 (5.55)	4.00%	Item 2	Items 2 & 6, 3 & 4

C	Items 1 & 2 sub-tested to account for dependency	35 (4.62)	–	Sub-test item 1 & 2	Items 3 & 4

D	Items 1 & 2 and 3 & 4 sub-tested to account for dependency	33 (4.36)	–	Sub-test item 1 & 2	None

E	Items 1, 2 & 6 and 3 & 4 sub-tested to account for dependency	27 (3.57)	–	Sub-test item 1, 2, 6	None

F	Item 2 removed and items 3 & 4 sub-tested to account for dependency	34 (4.49)	–	Item 1	None

G	Items 1 & 2 removed	20 (2.64)	–	Item 4 is borderline	Items 3 & 4

H	Items 1 & 2 removed and Items 3 & 4 sub-tested for dependency	20 (2.64)	–	None	None

PHQ2	PHQ-2 items only	6 (0.98)^c^	–	Item 2 (χ^2^ *P* = 0.0243)	None

a. Total number of tests 757.

b. Items that display response dependence at a residual correlation criterion value of 0.1.

c. Total number of tests 610.

#### Summary fit statistics

The summary statistics of the initial analysis (Table 1a, 1b) suggested some misfit within the scale as indicated by a significant χ^2^ item–trait interaction term and a high-item fit residual standard deviation. The series of *t*-tests suggest that the item set was not unidimensional; however, this can also be heavily influenced by the response dependency within an item set. This led to an exploration of the individual item fit.

The initial analysis fit statistics for each individual item are presented in Table 2. This indicates that items 1 and 2 are problematic in terms of the χ^2^ fit statistic and items 2 and 3 are problematic in terms of their fit residuals.

**Table 2:** Individual item fit for Patient Health Questionnaire-9 (PHQ-9) items

	Response category,^a^ *n* (%)				Logit location		Fit residual			χ^2^ probability
PHQ-9 item	0	1	2	3		s.e.		χ^2^	d.f.	
1 Little interest or pleasure in doing things	35 (4.6)	252 (32.9)	277 (36.1)	203 (26.5)	−0.353	0.051	−1.532	27.89	9	0.000

2 Feeling down, depressed or hopeless	22 (2.9)	201 (26.2)	301 (39.2)	243 (31.7)	−0.713	0.053	−3.697	60.166	9	0

3 Trouble falling or staying asleep, or sleeping too much	46 (6)	99 (12.9)	222 (28.9)	400 (52.2)	−0.582	0.048	2.952	13.172	9	0.154

4 Feeling tired or having little energy	23 (3)	121 (15.8)	264 (34.4)	359 (46.8)	−0.864	0.053	−1.447	16.895	9	0.050

5 Poor appetite or overeating	116 (15.1)	181 (23.6)	226 (29.5)	244 (31.8)	0.11	0.043	1.519	15.866	9	0.069

6 Feeling bad about yourself – or that you are a failure or have let yourself or your family down	58 (7.6)	185 (24.1)	252 (32.9)	272 (35.5)	−0.279	0.047	0.052	14.403	9	0.108

7 Trouble concentrating on things	90 (11.7)	229 (29.9)	234 (30.5)	214 (27.9)	0.076	0.045	0.896	10.542	9	0.308

8 Moving or speaking so slowly that other people could have noticed. Or the opposite – being so fidgety or restless that you have been moving around a lot more than usual	249 (32.5)	237 (30.9)	191 (24.9)	90 (11.7)	0.999	0.045	1.247	10.502	9	0.311

9 Thoughts that you would be better off dead or hurting yourself in some way	434(56.6)	195 (25.4)	93 (12.1)	45 (5.9)	1.606	0.048	0.972	20.037	9	0.017

a. Response categories: 0, not at all; 1, several days; 2, more than half the days; 3, nearly every day.

Item 2 appears to be the most problematic item. It displays a high-negative residual and an over-discriminating response pattern, indicating a possible redundancy or dependency within the item set.

#### Local independency

Two aspects of local independency were investigated. First the residual correlation matrix was assessed to identify response dependencies between items. At a correlation indication level of 0.1, dependencies were indicated between items 1 and 2 (*r* = 0.25), items 3 and 4 (*r* = 0.14) and items 2 and 6 (*r* = 0.17).

Second, the *t*-test results (Table 1a, 1b) indicated some evidence of multidimensionality, which could be caused by the response dependency found within the scale.

#### Targeting

**Fig. 1:**
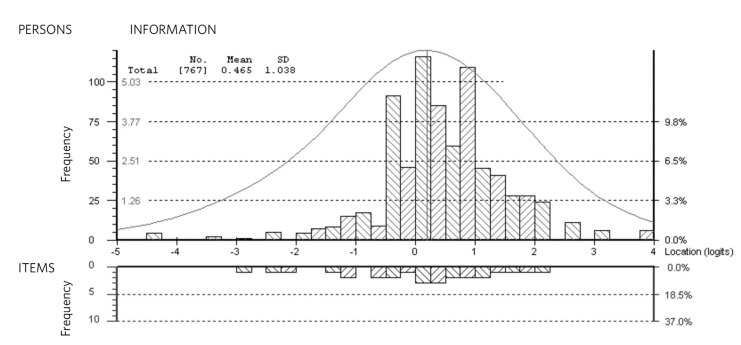
Person-item threshold distribution, displaying the relative logit location distribution of the PHQ-9 item thresholds and the primary care sample. The vast majority of the sample (91%) were classified as clinically depressed by the PHQ-9.

The person-item threshold distribution for the PHQ-9 scale is shown in Fig. 1. The scale appeared to be well targeted to this clinical sample, with the mean person location slightly higher than the mean item location. This indicates that this sample was displaying a higher average depression level than that represented by the scale (Fig. 1).

The results of the initial analysis show some potential problems with the scale among the current sample, although these problems are not entirely consistent with previous research in other populations.

### Part two: accounting for misfit within the model

The analyses iterations emphasise the problems that were apparent in the initial analysis (Table 1a, 1b). Thresholds remained ordered in all of the analyses, suggesting that all response categories are appropriate to this sample. The most parsimonious analysis iteration was analysis H, which has no specific problems despite the χ^2^ probability displaying significance. The overall significant χ^2^ value is likely to be affected by the reasonably large sample size. This is also the case for all of the other analyses, but individual problems were identified within analyses A–G.

For analysis H, when the overall χ^2^ value is adjusted based on a sample size of 500 (available within RUMM2030), *P* = 0.473. Within all analyses, once the response dependency has been taken into account the scale displays an acceptable level of unidimensionality. This suggests that the initial apparent multidimensionality is due to the response dependency that is present within the scale.

### PHQ-2

Items 1 and 2 were identified as displaying response dependency, and are potentially redundant when used in conjunction with all other PHQ-9 items. It should be noted that these two items make up the PHQ-2 short form. Additional analysis was carried out on the PHQ-2; results are reported in Table 1a and 1b.

Fit statistics displayed a degree of misfit, with the overall fit statistics similar to the better PHQ-9 analysis iterations. Also, to compare the PHQ-2 short form and the complete PHQ-9, individual person estimates were derived from each version of the scale (when anchored to the same metric). A *t*-test was then used to compare the PHQ-9 and PHQ-2 estimates for each individual. When test-based extremes are removed, 4 out of 757 (0.53%) individuals display person estimates that are significantly different at *P* = 0.05. When sub-test-based extremes are removed (i.e. those that were at the floor or ceiling of the shorter PHQ-2 scale), 2 out of 620 (0.32%) individuals display person estimates that are significantly different at *P* = 0.05. This same analysis also allows for equivalent levels of depression to be estimated on both the PHQ-9 and the PHQ-2. This revealed that the PHQ-9 cut-off point of 10 is equivalent to a PHQ-2 value of 2.705. When rounded to the nearest whole number, this is equivalent to the PHQ-2 cut-off point suggested by Kroenke *et al*^10^ as the optimal cut-off point for depression screening purposes.

These findings offer support for the use of PHQ-2 as a screening tool, as the person estimates of level of depression provided by the PHQ-2 do not significantly differ from the estimates provided by the PHQ-9.

## Discussion

The Rasch modelling process provides an integrated framework to explore different measurement characteristics of a scale. This integrated approach emphasises the relationship between the scale items and an assumed underlying latent construct. The Rasch model has a number of assumptions, including that of a unidimensional structure, which assumes that all of the items within a scale contribute to measuring the same underlying construct. Any deviation from this measurement structure will be identified through a series of fit statistics.^15^

Rasch analysis is a form of item response theory, as it is based around the interaction of how people respond to individual items within a scale. However, it is also often seen as separate entity owing to the differences in the epistemological approach. Andrich^26^ has broadly described these two approaches as the ‘statistical modelling’ paradigm (item response theory) and the ‘experimental measurement’ paradigm (Rasch), and has argued that the paradigms are incompatible, despite their apparent similarities. A distinctive feature of Rasch modelling is that the model is considered a formal representation of proper measurement and data are examined against this formal model, whereas with a statistical modelling approach the best model is sought to describe the data.^16^

To our knowledge this is the first attempt to apply Rasch models to a sample of primary care patients with varying levels of depression. Research in other areas has suggested different models for the PHQ-9, including the PHQ-2, which has been extensively developed to reduce the burden of time taken to identify people who may be experiencing depression.

The main finding of the study suggests that when all items of the PHQ-9 are taken together, then it contains items where response dependency is present among a sample with current depression. This is particularly apparent between items 1 and 2 and, to a lesser extent, between items 3 and 4. Both of these dependencies make sense conceptually, as the contents of items 1 and 2 are linked to the major symptoms of depression, whereas items 3 and 4 are concerned with issues of sleep and tiredness. It should be noted that items 1 and 2 are the two items that make up the PHQ-2 short form. This raises a question about the potential duplication of clinical information when items 1 and 2 are used alongside the other items within the PHQ-9.

Previous research^9^ found only three items of the PHQ-9 in their final solution: 1, 2 and 4. These core symptoms create the core ICD-10 criteria which link to the diagnosis of depression.^9^ Our research suggested that item 2 is over-discriminating and is potentially redundant in the existing scale. Conversely, this means that it could be seen as good summary item for the rest of the scale items, thus offering support for the use of the PHQ-2 as a screening tool. However, within the Rasch measurement model framework, the PHQ-2 items appear to be problematic when administered alongside the other items of the PHQ-9. The PHQ-2 has been advocated by some researchers as the preferred model of screening, with nurses reporting high satisfaction with an average screening process and reporting time of 1–2 min.^11^ Despite satisfaction on a practical clinical level, the sensitivity and specificity of the PHQ-2 with a sample of drug users in the community was shown to be poor in relation to the PHQ-9 in people with moderate clinical depression.^12^

The measurement properties of the PHQ-9 have also been explored by attempting to generate fit to the Rasch model through combinations of removal of mis-fitting items and sub-testing to account for dependency between items. This analysis procedure contributes towards the further understanding of the relationship between items of the scale. In this case, this analysis emphasised the findings of the initial analysis.

In summary, ordered response thresholds were never an issue: response dependency was apparent between the PHQ-2 items (items 1, 2), and between the sleep and tiredness items (items 3, 4). The initial apparent multidimensionality appears to be due to this dependency, and items 1 and 2 also overdiscriminate within the PHQ-9.

Our finding is contrary to that of previous research,^8^ which identified one mis-fitting item (item 8) that included contrasting symptoms. Williams *et al* argue that including both poles of the diagnostic criterion is confusing and is likely to contribute to item misfit. Consequently, they suggest that splitting items such as ‘poor appetite or overeating’ is likely to reduce cognitive demands, improve the psychometric properties, enhance specificity and minimise the costs associated with follow-up examinations of those who screen positive.^8^ However, from a psychometric point of view, splitting these items is likely to result in some dependency within the measure as the response to ‘poor appetite’ is unlikely to be independent from the response to ‘overeating’.

Overall, it is interesting to note that the four research studies using Rasch analysis have produced different models for the PHQ-9 with different populations. This point demonstrates the importance of validity within psychometric testing and variability of results across different groups of patients. For this reason, clinicians must weigh up the pros and cons of alternative cut-off points to determine the best fit for their circumstances.

In the present study, the most parsimonious analysis involved the removal of items 1 and 2, and accounting for the dependency between items 3 and 4. Following these amendments, the fit to the Rasch model appeared to be adequate. The scale appears to be well targeted to this particular sample, but the reported reliability values are not sufficient for the PHQ-9 to be used as an outcome measure for individual-level use. However, as the primary function of the PHQ-9 is as a screening tool rather than as an outcome measure, the reliability of the scale is probably sufficient.

### Study limitations

The sample of participants may not fully represent the diverse characteristics found within the wider population as patients with the most serious depression in this study are likely to be more severely impaired, the sample was screened into the study without external validation, and the purposive sampling may influence the findings of the analysis as a sample with depression would affirm items relating to symptomatic depression. Therefore, the apparent redundancy of the PHQ-2 items may be due to the sample inclusion criteria. Nonetheless, the conceptual redundancy still holds, regardless of the sampling. An improved strategy to assess the properties of the PHQ-9 would be to administer it to a clinically validated sample of patients with depression; however, it was beyond the scope of this study to collect the data in this way.

In its current format the PHQ-9 displays some problems with regard to its measurement structure among a sample with depression. However, these problems can be addressed to deliver a stable screening tool. The results also offer support for the PHQ-2 short form as a screening tool.
